# *Pseudomonas aeruginosa* GidA modulates the expression of catalases at the posttranscriptional level and plays a role in virulence

**DOI:** 10.3389/fmicb.2022.1079710

**Published:** 2023-01-16

**Authors:** Thanyaporn Srimahaeak, Narumon Thongdee, Jurairat Chittrakanwong, Sopapan Atichartpongkul, Juthamas Jaroensuk, Kamonwan Phatinuwat, Narumon Phaonakrop, Janthima Jaresitthikunchai, Sittiruk Roytrakul, Skorn Mongkolsuk, Mayuree Fuangthong

**Affiliations:** ^1^Program in Applied Biological Sciences, Chulabhorn Graduate Institute, Bangkok, Thailand; ^2^Department of Biotechnology, Faculty of Engineering and Industrial Technology, Silpakorn University, Sanamchandra Palace Campus, Nakhon Pathom, Thailand; ^3^Laboratory of Biotechnology, Chulabhorn Research Institute, Bangkok, Thailand; ^4^Functional Ingredients and Food Innovation Research Group, National Center for Genetic Engineering and Biotechnology, National Science and Technology Development Agency, Pathum Thani, Thailand; ^5^Center of Excellence on Environmental Health and Toxicology (EHT), OPS, MHESI, Bangkok, Thailand

**Keywords:** *Pseudomonas aeruginosa*, tRNA modification, oxidative stress, *gidA*, U34 modification, catalases, virulence

## Abstract

*Pseudomonas aeruginosa gidA*, which encodes a putative tRNA-modifying enzyme, is associated with a variety of virulence phenotypes. Here, we demonstrated that *P. aeruginosa gidA* is responsible for the modifications of uridine in tRNAs *in vivo*. Loss of *gidA* was found to have no impact on the mRNA levels of *katA* and *katB*, but it decreased KatA and KatB protein levels, resulting in decreased total catalase activity and a hydrogen peroxide-sensitive phenotype. Furthermore, *gidA* was found to affect flagella-mediated motility and biofilm formation; and it was required for the full virulence of *P. aeruginosa* in both *Caenorhabditis elegans* and macrophage models. Together, these observations reveal the posttranscriptional impact of *gidA* on the oxidative stress response, highlight the complexity of catalase gene expression regulation, and further support the involvement of *gidA* in the virulence of *P. aeruginosa*.

## 1. Introduction

*Pseudomonas aeruginosa* is a Gram-negative bacterium and one of the major causes of nosocomial infection in immunocompromised and weak patients (Nathwani et al., 2014; Moradali et al., 2017; Ciofu and Tolker-Nielsen, 2019). During *P. aeruginosa* infection, the first line of host defense involves the production of reactive oxygen and nitrogen species to kill the invading pathogen (Lavoie et al., 2011). Therefore, patients with deficient immune responses may not be able to cope with the infection leading to high mortality and morbidity rates (Lavoie et al., 2011). In 2017, the World Health Organization listed *P. aeruginosa* as a high priority organism for which new antibiotics are needed, as it poses a serious threat to human health (World Health Organization, 2017). *P. aeruginosa* infections are considered to have acute and chronic phases. A number of virulence factors are involved in the acute phase, including motility activity. Swimming motility is required for the initial step of the infection, wherein bacteria adhere to the host surface and expand their degree of infections. Meanwhile, in the beginning of a chronic infection, most activities related to virulence factors are reduced. Biofilm, one such virulence factor, plays a key role in developing multidrug resistance during this phase and thus leads to high mortality rates, especially among weak patients (Shi et al., 2019).

tRNA modifications are crucial for gene expression, particularly for the accuracy and efficacy of protein synthesis (de Crecy-Lagard and Jaroch, 2021). In addition to their global impact on tRNAs functions, many studies have demonstrated the regulatory role of tRNA modifications in controlling the translations of certain codon-biased mRNAs under specific conditions (Chan et al., 2018). In *Pseudomonas*, several tRNA-modifying genes have been shown to be associated with virulence gene expression or oxidative stress response (Kinscherf and Willis, 2002; Ahn et al., 2004; Gupta et al., 2009; Jaroensuk et al., 2016; Thongdee et al., 2019). GidA (also known as MnmE) and TrmE (also known as MnmG) together catalyze the incorporation of carboxymethylaminomethyl (cmnm) or aminomethyl (nm) into uridine (U) or 2-thio uridine (s^2^U) at the position 34 in the anticodons of tRNAs (Yim et al., 2006). Modifications formed by the GidA/TrmE complex are important for translation fidelity (Brégeon et al., 2001). The deletion of *gidA* affects a variety of phenotypes in several bacteria (Shippy and Fadl, 2014). In *Pseudomonas syringe*, *gidA* contributes to swarming motility and the production of lipodepsipeptide antibiotic and pyoverdine (Kinscherf and Willis, 2002). *gidA* also impacts the levels of LasA protease, rhamnolipid, and pyocyanin through the posttranscriptional control of the transcriptional regulator RhlR in the quorum sensing system of *P. aeruginosa* (Gupta et al., 2009). Furthermore, studies in other pathogenic bacteria have shown that a lack of *gidA* or *trmE* decreases the infection ability of *Salmonella enterica* serovar Typhimurium (Shippy et al., 2011), *Aeromonas hydrophila* (Sha et al., 2004), *Streptococcus pyogenes* (Cho and Caparon, 2008), and *Streptococcus suis* (Gao et al., 2020), suggesting GidA is a potential candidate for future vaccine development and a target for antibiotic development.

Although, the previous studies on *gidA* in *P. syringe* and *P. aeruginosa* have indicated the pleiotropic phenotype of *gidA* deletion, whether *gidA* influences *Pseudomonas* infection and how *gidA* controls gene expression remain unknown. Here, *gidA* was characterized *in vivo* as being required for the biosynthesis of cmcm^5^U and mnm^5^U tRNA modifications, revealing a novel role of *gidA* in the cellular response to hydrogen peroxide (H_2_O_2_) in *P. aeruginosa*. The impact of *gidA* deletion on biofilm formation that has not yet been linked with *Pseudomonas gidA* was examined in this study. In addition, a lack of GidA was found to attenuate *P. aeruginosa* virulence both *in vitro* and *in vivo*.

## 2. Materials and methods

### 2.1. Bacterial strains, plasmids, and cell lines

*Pseudomonas aeruginosa* strain UCBPP-PA14 (PA14) was used as the wild-type strain. All *Escherichia coli* and *Pseudomonas* strains were grown at 37°C in lysogeny broth (LB) or lysogeny agar (Lennox) unless otherwise indicated in the method. The following concentrations of antibiotic(s) were added: carbenicillin (Cb) at 200 μg/ml and gentamicin (Gm) at 75 μg/ml for *P. aeruginosa*; gentamicin (Gm) at 15 μg/ml and ampicillin (Ap) at 100 μg/ml for *E. coli*. All bacterial strains and plasmids used in this study are listed in Table 1.

**Table 1:** Strains and plasmids used in this study.

**Strain/plasmid**	**Characteristic**	**Reference/source**
*P. aeruginosa* PA14		
Wild type	*P. aeruginosa* UCBPP-PA14 wild-type strain	ATCC
*gidA* mutant	*gidA*::pKNOCK*-*Gm,Gm^r^	This study
*gidA* complemented	*gidA* mutant harboring pBBR1MCS-4/*gidA*_FL	This study
*E. coli*		
BW20767	*leu-63*::IS*10recA1creC510 hsdR17 endA1zbf-5uidA*(∆*MluI*)::pir^+^*thi* RP4-2-tet::Mu-1kan::Tn7	Metcalf et al., 1996
DH5α	λ^−^ ϕ80d*lacZ*∆M15 r*ecA*1 *endA*1*gyrA96thi-1hsdR17*(r_K_^−^m_K_^+^) *supE44relA1deoR* ∆(*lacZYA*-*argF*)*U169*	Stratagene Inc. (United States)
DE3 (BL21)	*E. coli* B F- *dcm ompT hsdS* (r_B-_m_B-_) *galλ* (DE3)	Stratagene Inc. (United States)
Plasmids		
pKNOCK-Gm	Suicide plasmid for insertional inactivation of *gidA*	Alexeyev, 1999
pKN-*gidA*	pKNOCK-Gm with internal 215-bp fragment of *gidA*	This study
pBBR1MCS-4	Broad-host-range plasmid	Kovach et al., 1995
pBB-*gidA*-FL	pBBR1MCS-4 expressing PA14 GidA	This study
pBB-*katB*-6Xhis	pBBR1MCS-4 expressing His-tagged KatB	This study

### 2.2. Construction of the *gidA* mutant strain and *gidA* complemented strain

A *gidA* mutant strain was constructed using the pKNOCK suicide vector (Alexeyev, 1999). A 200 bp *gidA* fragment was amplified from the genomic DNA of *P. aeruginosa* PA14 using polymerase chain reaction (PCR) with the primer pair BT4409 (5′-CACATCGGCCTGGAGAAC-3′) and BT4410 (5′-GAACCGAGGAAGGACATCAC-3′). The amplified fragment was ligated into the *Sma*I site of the pKNOCK vector, which carried a Gm^R^ cassette (pKNOCK-Gm^R^). The ligation reaction was transformed into *E. coli* strain BW20767, which was screened for the gentamicin resistance phenotype (Metcalf et al., 1996). The resulting plasmid was named pKN-*gidA* and transferred to the recipient *P. aeruginosa* PA14 by biparental mating using *E. coli* BW20767 as the donor strain. The *gidA* mutant was verified using colony PCR and confirmed using Southern blot analysis.

A full-length DNA fragment of the *gidA* gene was amplified from PA14 using primers BT5113 (5′-CCGAGGTGCGTGGTGGATT-3′) and BT5114 (5′-TGGGTTACCGCAGACATCAAG-3′). The *gidA* full-length DNA fragment was ligated into the *Sma*I restriction site of the pBBR1MCS-4 vector (Kovach et al., 1995), which contains an Amp^R^ cassette, to produce a pBB-*gidA* plasmid. To obtain the *gidA* complemented strain, the verified pBB-*gidA* was transformed into a *gidA* mutant by electroporation. The *gidA* complemented strain was then screened on LB agar plates containing carbenicillin and gentamicin and confirmed using colony PCR analysis.

### 2.3. Ribonucleoside analysis

Total tRNA *in vivo* was purified as previously described (Jaroensuk et al., 2016; Thongdee et al., 2019). Total RNA was purified from logarithmic phase cultures using Trizol reagent (Invitrogen). Large RNA species were separated from small RNA species using 35% ethanol precipitation. tRNA was purified from the small RNA pool using size-exclusion high performance liquid chromatography (SEC-HPLC) with an Agilent SEC3 300 Å, 7.8 × 300 mm column. The SEC-HPLC was operated under isocratic elution mode using 100 mM ammonium acetate as the mobile phase. The concentration and quality of total tRNA were assessed using a bioanalyzer (Agilent Technologies). To produce ribonucleoside products, the purified tRNA was treated with benzonase nuclease (Sigma), bacterial alkaline phosphatase (Invitrogen), and phosphodiesterase in the presence of deaminase inhibitors (0.5 μg/ml coformycin and 5 μg/ml tetrahydrouridine) and antioxidants (50 μM desferrioxamine and 50 μM butylated hydroxytoluene) at 37°C overnight. The ribonucleosides were fractionated using reverse phase chromatography (Thermo Hypersil Gold a Q column, 100 × 2.1 mm, 1.9 μm particle size) with a gradient of 0.1% (v/v) formic acid in water (solvent A) and 0.1% (v/v) formic acid in acetonitrile (solvent B). The HPLC column was directly connected to a triple quadrupole mass spectrometer (Agilent LC/QQQ 6490) operated in the positive ion mode. Ribonucleosides were identified by comparing their *m/z*, CID fragmentation patterns, fragmentor voltages, collision energies, and HPLC retention times with chemical synthetic standards.

### 2.4. Minimum inhibitory concentration (MIC) assay

MIC assays were performed to determine the minimum inhibitory concentration of H_2_O_2_ - that is, the lowest concentration that inhibits bacterial growth (Wiegand et al., 2008). Briefly, an overnight cell culture was diluted in Mueller Hinton Broth (MHB) and grown to a turbidity of 0.5 McFarland standard. The culture was then diluted by a factor of 1:20 with 0.9% sterile normal saline. The diluted culture was used to inoculate each well of a 96-well plate containing different concentrations of a test chemical solution prepared in MHB. MHB without cells were used as a negative control, while MHB with cells inoculated but without any test chemical was used as a positive control. The 96-well plate was incubated at 37°C. After 16–20 h, the turbidity level in each well was measured using a microplate reader.

### 2.5. Catalase activity assay

Exponential phase cultures were treated with or without H_2_O_2_ at 5 mM or 10 mM for 25 min at 37°C with shaking. The cell pellets were washed three times with 50 mM phosphate buffer at pH 7.4 before being subjected to sonication. Crude protein concentration was then measured using the Bradford assay. Specific catalase activity (U/mg protein) was spectrophotometrically measured (A_420_, light path = 1 cm). U was defined as one unit of catalase decomposing 1.0 μmole of H_2_O_2_ per minute at pH 7.0 at 25°C. 10 mM H_2_O_2_ in 50 mM phosphate buffer (pH 7.0) was used as the substrate.

### 2.6. Western blot analysis

Exponential phase cultures were treated with or without 5 mM H_2_O_2_ for 25 min at 37°C with shaking. 50 μg of crude protein was separated using 12.5% SDS-PAGE. The separated proteins were transferred onto the polyvinylidene difluoride (PVDF) membranes (GE Healthcare). The membranes were blocked with 5% skim milk in TBST buffer (20 mM Tris–HCl pH 7.5, 150 mM NaCl, and 0.05% Tween 20) for at least 1 h before incubating with antibody solution (horse radish peroxidase-conjugated anti-His tag antibody diluted in 3% skim milk and 1% BSA in TBST) for 1 h. The membranes were washed three times for 30 min in TBST buffer. The antibody reaction was observed using a chemiluminescent (ECL) detection reagent kit (GE Healthcare) according to the company’s recommendation. KatA and KatB were expressed from the pBB-*katA*-6xHis plasmid and the pBB-*katB*-6xHis plasmid, respectively. The pBB-*katB* 6XHis plasmid was previously described (Thongdee et al., 2019). pBB-*katA* 6XHis plasmid was constructed in a similar fashion to the pBB-*katB* 6XHis plasmid. The full-length *katA* gene was amplified from PA14 chromosomal DNA using PCR with the primer pair BT5117 (5′-GTTCTCCGTGGTCGCCC-3′) and BT6458 (5′-TTAGTGGTGGTGGTGGTGGTGGTCCAGCTTCAGGCCGAGG-3′), which contains hexahistidine (6XHis) tag. The *katA*-6XHis fragment was ligated into the *Bam*HI and *Eco*ICRI restriction sites of the pBBR1MCS-4 vector (Kovach et al., 1995) resulting in pBB-*katA* 6XHis plasmid for gene expression in PA14. The verified pBB-*katA* 6XHis plasmid was introduced into the wild-type and *gidA* mutant strains for Western blot analysis.

### 2.7. Semiquantitative real-time PCR

Exponential phase cultures grown in LB medium and treated with or without 5 mM H_2_O_2_ for 10 min at 37°C were used to determine the level of gene expression. A hot acid phenol extraction protocol was used for total RNA extraction (Chomczynski and Sacchi, 1987). Following DNase treatment, cDNA was synthesized using random hexamer primers and a RevertAid^™^ M-MuLV Reverse Transcriptase Kit (Thermo Fisher Scientific). Semiquantitative real-time PCR was performed using KAPA SYBR FAST reagent (KAPA Biosystems) and a StepOnePlus^™^ real-time PCR system (Thermo Fisher Scientific) according to the manufacturers’ instructions. The specific primers for the genes of interest were as follows: BT5637 (5′-TCTCCATGCGTTTCTACACC-3′) and BT5638 (5′-CGCATTGATGAAGCTGAAGG-3′) for *katA*; BT5639 (5′-CGACGCTTCGATTTCTTCTC-3′) and BT5640 (5′-TTCGGATCGAGGTTCTTCTG-3′) for *katB*; BT5641 (5′-CTACGGCGAGTTCCAGAAAG-3′) and BT5642 (5′-AGTGGATCTCGACGGTCTTG-3′) for *ahpC*; BT8259 (5′-CGCGAAGAAATACGACGCCG-3′) and BT8260 (5′-GTCGCTGAGGATGCCGTAGTA-3′) for *fliA*; BT8261 (5′-AAGGTCGAGGTCAGCGATGAC-3′) and BT8262 (5′-TCACCACGGTCTGTTCGTTGAT-3′) for *fliD*; BT8263 (5′-AACCCGCACCGTCTGATCC-3′) and BT8264 (5′-GCCTCGACCAGACGAGCGA-3′) for *fliS*; BT8253 (5′-TGGACAAGCAGACCGGCGAC-3′) and BT8254 (5′-TACATCCTTGGGCAGGCAGG-3′) for *algD*; BT8255 (5′-GGTGCCGCTCATCGTGCTCTA-3′) and BT8256 (5′-CGCGTTGCCCTGCATCTGGTA-3′) for *algK*; BT8257 (5′-CTACCCTGCCGAAGCTGGAT-3′) and BT8258 (5′-TCGCTGGACGAGGAGTTGGT-3′) for *mucA*; and BT2781 (5′-GCCCGCACAAGCGGTGGAG-3′) and BT2782 (5′-ACGTCATCCCCACCTTCCT-3′) for 16 s RNA gene. The 16S ribosomal RNA gene was used as the internal control to normalize gene expression.

### 2.8. Motility assays

Motility was examined on BM2-swimming plates (0.3% bacto agar, 62 mM potassium phosphate buffer pH 7, 7 mM (NH_4_)_2_SO_4_, 2 mM MgSO_4_, 10 μM FeSO_4_, 0.4% glucose) and BM2-swarming plates (0.5% bacto agar, 62 mM potassium phosphate buffer pH 7, 0.1% casamino acid, 2 mM MgSO_4_, 10 μM FeSO_4_, 0.4% glucose; Overhage et al., 2007, 2008; Filloux and Ramos, 2014). Homogenized colonies were stabbed into the center of the BM2-swimming plate while 5 μl of the overnight cultures was inoculated on the surface of each BM2-swarming plate. The swimming and swarming plates were incubated overnight at 37°C. The relative motility of each strain was determined by comparing the diameters of the swim and swarm colonies.

### 2.9. Biofilm formation assay

Two methods were used to observe the biofilm formation, i.e., dye straining and scanning electron microscope (SEM). For the dye staining method, 100 μl of a diluted overnight culture (OD_600nm_ ~ 0.2) was added to a well of a 96-well plate and incubated overnight at 37°C. After overnight incubation, the plate was gently rinsed with distilled water before staining with 150 μl of 1% crystal violet for 10 min. Then, the crystal violet solution was removed. The plate was rinsed with distilled water again and allowed to air dry for at least 15 min. 100 μl of absolute ethanol was added into the well to dissolve the remaining crystal violet. Then, a spectrophotometer measurement was conducted at an optical density of 600 nm. The SEM method was performed using the method of Jin et al. with some adaptations (Jin et al., 2005). The biofilm of wild-type, *gidA* mutant, and complemented strains were developed on 1 × 1 cm plastic slide. The plastic slides were pretreated with concentrated sulfuric acid and 95% ethanol. 100 μl of cell suspensions (10^7^ CFU/ml) in PBS were dropped on the slide and were incubated for 90 min at 37°C to promote the bacterial cell adhesion. The plastic slides were washed twice with 200 μl PBS to remove nonadherent cells and filled with 100 μl Brain Heart Infusion (BHI) culture medium. The bacterial cultures were incubated at 37°C for 36 h and the BHI culture medium was replenished daily. The biofilm samples were fixed with 2.5% glutaraldehyde for 2 h and serially dehydrated with ethanol (70%, 95%, and absolute ethanol). The samples were kept in absolute ethanol until analysis. Before observing under the SEM, all samples were dried using critical-point drying in an EMITECH K850 dryer (Quorum Technologies Ltd.) at 39.1°C and 1,000 psi. The dried samples were then coated with gold, and the images were obtained using a TESCAN MIRA3 FEG-SEM (Field Emission Scanning Electron Microscope).

### 2.10. Alginate measurement

Alginate measurement was adapted from methods of Knutson and Jeanes (1968) and Zheng et al. (2016). Wild type, *gidA* mutant and the complement strain were spread onto BHI agar to promote alginate production. The cells were incubated at 37°C for 40–48 h, harvested and resuspended in 5 ml PBS (pH 7.4). Approximately 10^7^ cells were centrifugation at 12,000 g for 20 min to remove the cell pellet. Alginate was precipitated by adding equal volume of 2% cetylpyimidine to the cell free medium followed by centrifugation at 12,000 g for 15 min. The pellets were resuspended in 5 ml of 1 M NaCl. Then, 5 ml ice-cold isopropanol (−20°C) was added before centrifugation at 12,000 g for 15 min to collect the alginate pellet. Completely air-dried alginate pellets were then resuspended in 1 ml 0.9% NaCl. Measurement of alginate in the solutions using the standard alginic acid (0.01–1.0 mg/ml in 0.9% NaCl) was performed in 1.5 ml microtubes. 70 μl of alginate suspension was mixed using vortex with freshly prepared ice-cold 0.1 M H_3_BO_3_ in concentrated H_2_SO_4_. Then, 20 μl of 0.1% carbazole in ethanol was added. The solution was mixed and incubated at 55°C for 30 min. The absorbance at 530 nm was measured. The amount of alginate in each sample was calculated against alginate standards and normalized with OD_600_ of the bacterial cell suspension.

### 2.11. *In vitro* phagocytosis assay

The phagocytosis assay was modified from previously described protocols (Kuang et al., 2011; Charoenlap et al., 2012; Gao et al., 2016). Macrophage cells (RAW264.7) were activated with mouse IFN-gamma. Activated macrophage cells (RAW264.7) were seeded (2 × 10^6^ cells/well) in six-well tissue culture plates overnight. The cells were infected with mid-log phase *P. aeruginosa* cultures (MOI 1:10). The live and dead macrophage cells were counted at 0, 2, 4, and 8 h.

### 2.12. *Caenorhabditis elegans* killing assay

The virulence of *P. aeruginosa* was tested using *Caenorhabditis elegans* as the animal model (Tan et al., 1999). *P. aeruginosa* strains were grown in 5 ml of King’s broth at 37°C overnight with shaking. 10 μl of each overnight culture was spread onto high osmotic strength PGS agar (peptone-glucose-sorbital; 1% Bacto-peptone, 1% NaCl, 1% glucose, 0.15 M orbital, 1.7% Bacto agar) in a 60 × 15 mm plate. The plates were incubated overnight at 37°C and then subsequently transferred to 22°C for 8–12 h. Each bacterial lawn plate was seeded with 20–200 L4 stage larvae of *C. elegans* (Bristol-N2 wild-type strain from BP. Braeckman’s lab), incubated at room temperature, and scored for live and dead worms at time intervals over 3 days.

### 2.13. Total shotgun proteome analysis

The exponential phase cells were snap-frozen in liquid nitrogen. Total protein was isolated using 0.5% SDS solution. Protein content was measured using the Lowry assay with bovine serum albumin as a standard (Lowry et al., 1951). 5 μg of protein sample was subjected to in-solution digestion. All samples were completely dissolved in 10 mM ammonium bicarbonate (AMBIC). Disulfide bonds were reduced using 5 mM dithiothreitol (DTT) in 10 mM AMBIC at 60°C for 1 h. Alkylation of sulfhydryl groups was done by incubating them with 15 mM iodoacetamide (IAA) in 10 mM AMBIC at room temperature for 45 min in the dark. Then, the samples were mixed with 50 ng/μL of sequencing grade trypsin (1:20 ratio; Promega, United States) and incubated at 37°C overnight. The digested samples were dried and redissolved with 0.1% formic acid before injection into an Ultimate3000 Nano/Capillary LC System (Thermo Scientific, United Kingdom) coupled to an HCTUltra LC–MS system (Bruker Daltonics Ltd.; Hamburg, Germany) equipped with a nano-captive spray ion source. 5 μl of peptide digest was enriched on a μ-Precolumn 300 μm i.d. × 5 mm C18 Pepmap 100, 5 μm, 100 A (Thermo Scientific, United Kingdom), separated on a 75 μm i.d. × 15 cm and packed with Acclaim PepMap RSLC C18, 2 μm, 100 Å, nanoViper (Thermo Scientific, United Kingdom). The C18 column was enclosed in a thermostatted column oven set at 60°C. Solvents A and B containing 0.1% formic acid in water and 0.1% formic acid in 80% acetonitrile, respectively, were used as mobile phases. A gradient of 5–55% solvent B was used to elute the peptides at a constant flow rate of 300 nl/min for 30 min. Electrospray ionization was carried out at 1.6 kV using CaptiveSpray. Nitrogen was used as a drying gas at a flow rate of about 50 l/h. Collision-induced dissociation (CID) product ion mass spectra were obtained using nitrogen gas as the collision gas. Mass spectra (MS) and MS/MS spectra were obtained in the positive-ion mode at 2 Hz over a range of 150–2,200 *m/z*. The collision energy was adjusted to 10 eV as a function of the *m/z* value. Liquid chromatography-mass spectrometry (LC–MS) analysis of each sample was done in triplicate. The raw MS/MS spectra data are available in ProteomeXchange: JPST001884 and PXD037219.^1^

### 2.14. Bioinformatics and shotgun proteome data analysis

For protein quantitation, DeCyder MS Differential Analysis software (DeCyderMS, GE Healthcare) was used (Johansson et al., 2006). The analyzed MS/MS data from DeCyderMS were submitted for database searches using Mascot software (Matrix Science, London, United Kingdom). The data were searched against the NCBI database for protein identification. Database interrogation was performed for taxonomy (*Pseudomonas aeruginosa*); enzyme (trypsin); variable modifications (carbamidomethyl, oxidation of methionine residues); mass values (monoisotopic); protein mass (unrestricted); peptide mass tolerance (1.2 Da); fragment mass tolerance (±0.6 Da); peptide charge state (1+, 2+ and 3+); and missed cleavages. The level of protein in each sample was expressed as a log2 value. Gene ontology annotation was performed using Panther (Mi et al., 2019).

## 3. Results

### 3.1. *Pseudomonas aeruginosa* GidA is a tRNA-modifying enzyme in the mnm^5^(s^2^)U biosynthetic pathway

**Figure 1:**
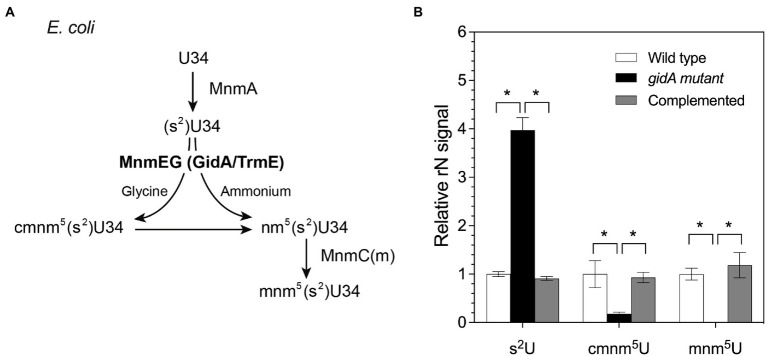
The amount of tRNA modifications in the mnm^5^(s^2^)U biosynthetic pathway is altered in the *gidA* mutant. **(A)** The pathway of mnm^5^(s^2^)U biosynthesis in *E. coli*. **(B)** Change in the levels of s^2^U, cmnm^5^U, and mnm^5^U in total tRNA isolated from wild-type, *gidA* mutant, and complemented strains. The wild type and *gidA* mutant carried the pBBR1MCS-4 plasmid as a vector control. The level of each modified ribonucleoside was quantified based on the MRM signal intensity and normalized by dividing the quantified amounts by the summed signals of adenosine, guanosine, cytidine, and uridine in the sample. The data represent the mean ± SD values of three biological replicates. The statistical analysis was performed using Graphpad Prism (GraphPad Software). Asterisks denote a significant difference in the one-way analysis of variance (ANOVA; * *p*≤ 0.05).

*P. aeruginosa* GidA displays 70% identity to the *E. coli* MnmE protein (Yim et al., 2006). To assess whether *P. aeruginosa* GidA is responsible for the formation of cmnm^5^(s^2^)U or nm^5^(s^2^)U in the mnm^5^(s^2^)U biosynthetic pathway (Figure 1A), tRNA modification profiles of the wild type, a *gidA* insertional inactivation mutant, and a complemented strain were analyzed using LC–MS/MS. The result of this study showed that the inactivation of *gidA* specifically altered the level of tRNA modifications linked to the mnm^5^(s^2^)U biosynthetic pathway (Figure 1B). Among the 24 modifications detected in *P. aeruginosa*’s tRNAs, only the s^2^U, cmnm^5^U, and mnm^5^U levels were found to change due to the loss of *gidA* (Table 2; Figure 1B). The s^2^U levels increased around fourfold, while the cmnm^5^U and mnm^5^U levels significantly reduced in the *gidA* mutant compared to those in the wild-type and complemented strains (Figure 1B; Table 2). These findings denote the crucial function of *gidA* in the biosynthesis of 5–carboxymethylaminomethyl uridine (cmnm^5^U) and 5-methylaminomethyl uridine (mnm^5^U) tRNA modifications in *P. aeruginosa*. In addition, hypomodifications of cmnm^5^U and mnm^5^U also resulted from the disruption of *P. aeruginosa trmE* (Supplementary Figure S1), indicating that the formation of cmnm^5^U and mnm^5^U is dependent on *gidA* as well as *trmE*.

**Table 2:** Ribonucleotides identified by LC–MS/MS analysis of *P. aeruginosa* PA14 tRNA.

**rN Name**	**Precursor ion *(m/z*)**	**Product ion (*m/z*)**	**Fragment (V)**	**Collision (*V*)**	**Retention time (min)**	**Fold change in ribonucleotide synthesis**					
						***gidA* mutant/Wild type**		***gidA* complemented/Wild type**		***gidA* mutant/*gidA* complemented**	
						**Mean**	**SD**	**Mean**	**SD**	**Mean**	**SD**
rC	244.1	112.1	380	8	1.6	1.02	0.02	1	0.02	1.01	0.01
rU	245.1	113.1	380	4	2.6	0.98	0.02	0.98	0.01	1	0.01
Ψ	245.1	125.1	380	8	1.44	0.98	0.04	0.98	0.04	1	0.02
D	247.1	115.1	380	8	1.41	1.04	0.05	1.05	0.05	0.99	0.03
Cm	258.1	112.1	380	8	5.1	1.17	0.09	1.18	0.07	0.99	0.06
m^5^C	258.1	126.1	380	8	2.7	0.96	0.31	1.3	0.41	0.74	0.05
m^3^C	258.1	126.1	380	8	2.2	0.81	0.57	0.94	0.61	0.81	0.18
Um	259.1	113.1	380	4	9.1	3.06	1.63	3.06	1.5	1	0.15
m^5^U	259.1	127.1	380	8	6.3	0.94	0.05	0.95	0.07	0.99	0.04
S^4^U	261.1	129.1	380	8	7.9	0.92	0.13	0.97	0.17	0.96	0.08
S^2^U	261.1	129.1	380	8	6.3	3.97*	0.26	0.91	0.04	4.37*	0.11
rA	268.1	136.1	380	16	5.5	1	0	1	0	1	0
I	269.1	137.1	380	12	6.2	0.84	0.03	0.87	0.06	0.96	0.04
mo^5^U	275.1	143.1	380	4	7.3	0.79	0.19	0.88	0.28	0.93	0.16
m^6^A	282.1	150.1	380	16	16.2	0.93	0.04	0.87	0.03	1.06	0.08
m^2^A	282.1	150.1	380	16	11.5	0.96	0.06	0.95	0.08	1.01	0.02
Am	282.1	136.1	380	16	12.4	1.07	0.42	1.03	0.37	1.11	0.42
rG	284.1	152.1	380	16	6.9	1	0.01	1	0.01	1	0.01
mnm^5^U	288.1	125	380	20	1.6	0.06*	0.01	1.15	0.19	0.05*	0
m^6^_2_A	296.1	164.1	380	16	20.4	0.5	0.25	1.12	0.41	0.44*	0.07
Gm	298.1	152.1	380	8	15	0.99	0.2	0.94	0.23	1.06	0.05
m^7^G	298.1	166.1	380	12	5.1	0.91	0.02	0.95	0.02	0.96	0.03
m^1^G	298.1	166.1	380	12	15	0.99	0.24	0.9	0.16	1.11	0.26
m^2^_2_G	312.1	180.1	380	8	19.5	0.47	0.4	0.75	0.53	0.57	0.13
cmo^5^U	319	187	380	8	6.5	0.78	0.09	0.86	0.08	0.91	0.03
cmnm^5^U	332.1	125	380	18	1.6	0.18*	0.03	0.93	0.11	0.19*	0.02
i^6^A	336.2	204.2	380	16	22.4	0.74	0.23	0.67	0.19	1.1	0.19
cmnm^5^s^2^U	348.1	141.1	380	20	3.7	0.53	0.75	1.11	0.73	0.35	0.33
t^6^A	413.1	281.1	380	8	21.9	0.91	0.04	0.92	0.03	0.99	0.02

Asterisks denote a significant difference in the one-way ANOVA (*p ≤ 0.05).

### 3.2. Loss of *gidA* decreases the expression of *katA* and *katB* at the posttranscriptional level leading to H_2_O_2_ susceptibility in *Pseudomonas aeruginosa*

Cell response to oxidative stress is an important mechanism of *Pseudomonas* for counteracting the toxic effects of oxidizing agents such as hydrogen peroxide, superoxide, and hypochlorous acid, which are produced by the host immune system during infection. In our H_2_O_2_ sensitive phenotype screening from the mutant library of *Pseudomonas aeruginosa*, *gidA* mutant is one of mutant strains showing such phenotype (unpublished data). To further determine the role of *gidA* in H_2_O_2_ stress response, the sensitivity of a *gidA* mutant strain to H_2_O_2_ was tested using a minimal inhibition concentration (MIC) assay. The results revealed that the *gidA* mutant (78 nM) was more susceptible to H_2_O_2_ than the wild-type and complemented strains (156 nM). The same result was found in the experiment involving the use of a plate sensitivity assay (data not shown). Therefore, it is possible that *gidA* is necessary for full resistance against H_2_O_2_.

**Figure 2:**
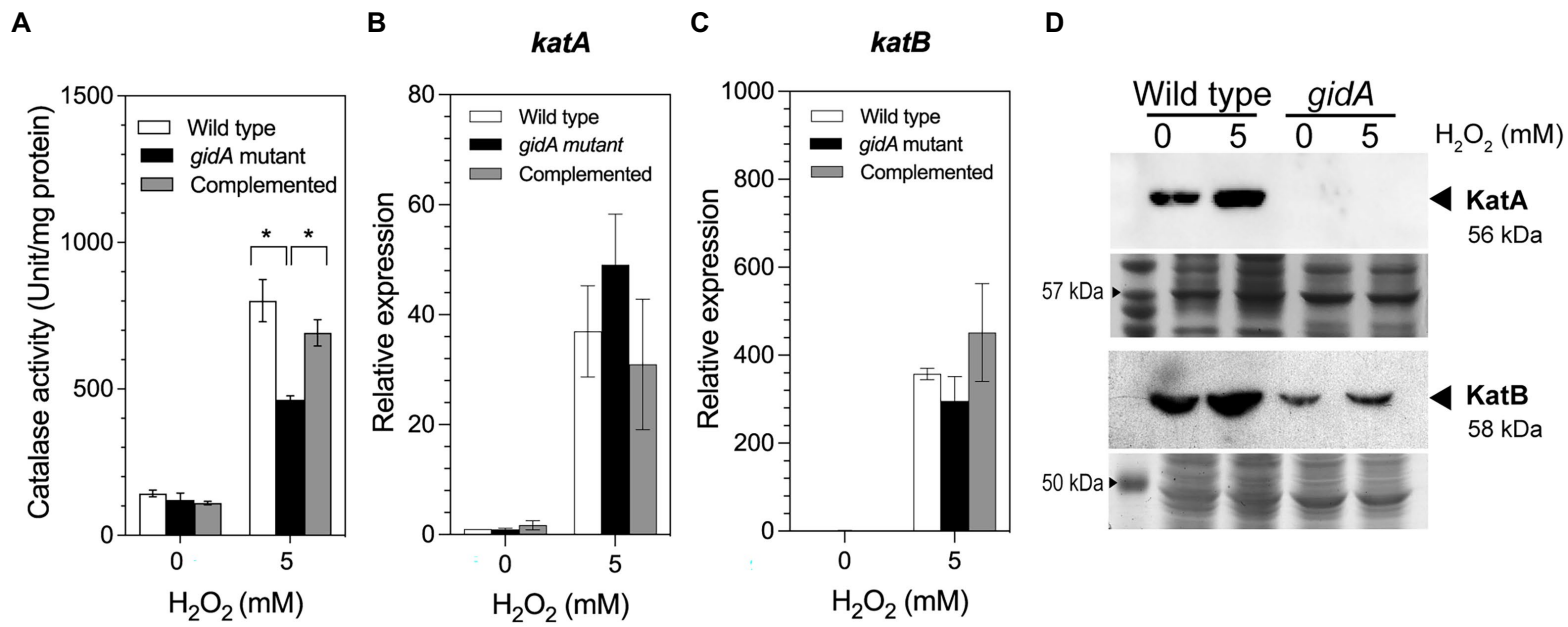
Impact of *gidA* disruption on catalase gene expression. **(A)** The catalase-specific activity of the wild-type, *gidA* inactivation mutant, and complemented strains. **(B)** Relative mRNA expression levels of *katA*, and **(C)** *katB*, in the wild-type, *gidA* mutant, and complemented strains upon H_2_O_2_ exposure. The wild-type and *gidA* mutant carried the pBBR1MCS-4 plasmid as a vector control. The data represent the mean ± SD values of three biological replicates for **(A–C)**. The statistical analysis was performed using Graphpad Prism (GraphPad Software). Asterisks denote a significant difference in the one-way ANOVA (* *p* ≤ 0.05). **(D)** Western blot analysis of the KatA and KatB proteins. Anti-KatA antibody was used to assess KatA level. The amounts of KatA and KatB proteins overexpressed from the pBB-*katA*-6xHis plasmid and pBB-*katB*-6xHis plasmids, respectively, were assessed using the anti-His antibody. The amount of total protein loaded in each gel is shown as the loading control. The data shown are representative results of three biological replicates.

The detoxification of H_2_O_2_ into harmless H_2_O and O_2_ molecules is primarily catalyzed by catalase enzymes such as KatA and KatB (Zamocky et al., 2010). Disruption of *gidA* may affect the capability of *P. aeruginosa* to detoxify H_2_O_2_. To test this hypothesis, the total catalase activity of the *gidA* mutant, the wild-type, and complemented strains were compared. Under unexposed conditions, the total catalase activities were not statistically different (143.3 ± 11.1, 121.6 ± 23.0, and 109.3 ± 5.8 units/mg protein, respectively; Figure 2A). Upon exposure to 5 mM H_2_O_2_, the total catalase activities of all strains increased from the basal level (Figure 2A). However, the catalase activity of the *gidA* mutant (462.9 ± 13.4 units/mg protein) was significantly lower than those of the wild-type (801.1 ± 72.1 units/mg protein) and complemented strains (690.6 ± 45.2 units/mg protein). This observation shows that the disruption of *gidA* has a negative effect on catalase-mediated H_2_O_2_ detoxification in *P. aeruginosa*. The reduced catalase activity of the *gidA* mutant may not be sufficient for cell survival during H_2_O_2_ treatment, resulting in a higher H_2_O_2_ sensitivity of the *gidA* mutant than those of the wild-type and complemented strains.

*P. aeruginosa* possesses three catalase enzymes; KatA, KatB, and KatE. To further explain how the loss of *gidA* affects catalase activity, the impact of *gidA* disruption on catalase gene regulation was determined. The regulation of *P. aeruginosa katA* and *katB*, which encode major catalases expressed during exponential phase growth, was investigated. The results of a semiquantitative real-time PCR assay demonstrated that the transcript levels of *katA* and *katB* increased in response to H_2_O_2_ exposure in all strains, as expected, and that the transcript levels were not statistically different. Moreover, no significant differences in transcript levels were observed between the strains in the absence of H_2_O_2_ (Figures 2B,C).

Given that total catalase activity was significantly reduced due to the absence of *gidA* (Figure 2A), it is thus highly possible that *gidA* exerts its effect on *katA* and *katB* gene expression at the posttranscriptional level. Western blot analysis was used to investigate the impact of *gidA* on the protein level of KatA and KatB during exposure to 5 mM H_2_O_2_. We investigated the level of KatA and KatB expressed from the plasmid copy, where gene is expressed using the *lac* promoter and ribosome binding site from the plasmid. The result demonstrated that KatA protein expression was not detectable in the *gidA* inactivation mutant compared to the wild-type strain under both normal and 5 mM H_2_O_2_ treatment conditions (Figure 2D). In addition, the level of KatB protein was markedly decreased in the *gidA* mutant compared with that in the wild type (Figure 2D). The decreased level of both KatA and KatB protein, but not *katA* and *katB* mRNA level, indicates that *gidA* exerts a posttranscriptional effect on *katA* and *katB* expression.

### 3.3. The *gidA* disruption has a negative effect on motility and biofilm formation in *Pseudomonas aeruginosa*

**Figure 3:**
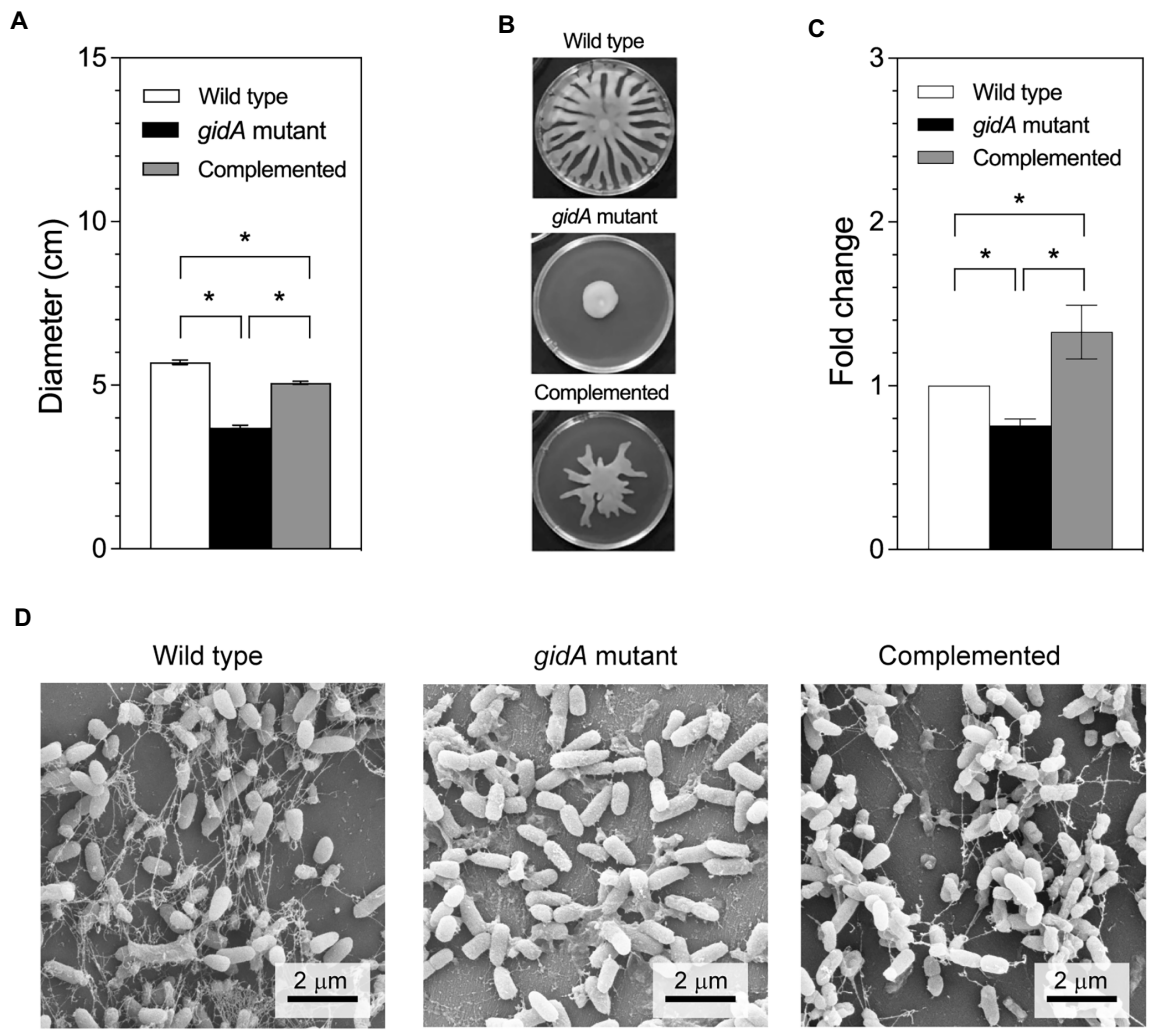
The *gidA* mutant strain shows defects in motility and biofilm production. **(A)** Swim colony diameter. Bacteria were stabbed into 0.3% BM2 medium agar. After incubation, the diameter of bacterial migration was measured from the stab point. **(B)** Images of the swarming plates. **(C)** The fold change in biofilm production for the wild-type, *gidA* mutant, and complemented strains using dye staining method. **(D)** Scanning electron microscope showing the biofilm formation. The images were recorded at magnification of 15,000x. The wild type and *gidA* mutant carried the pBBR1MCS-4 plasmid as a vector control. The data represent the mean ± SD values of three biological replicates for **(A,C)**. The statistical analysis was performed using Graphpad Prism (GraphPad Software). Asterisks denote a significant difference in the one-way ANOVA (* *p* ≤ 0.05).

It is known that *gidA* is associated with virulence mechanisms and the expression of a wide range of virulence genes in bacteria, including quorum sensing and stress responses (Gupta et al., 2009; Shippy and Fadl, 2014; Zhang et al., 2014; Gao et al., 2016). In *P. aeruginosa* PA14, *gidA* is involved in the Rhl-quorum sensing system affecting the bacterium’s growth and proteolytic activities (Gupta et al., 2009). Therefore, genes associated with bacterial motility and biofilm formation assays were performed in this study. Figures 3A,B present the results for both motility activities, i.e., swimming and swarming. With respect to swimming motility (Figure 3A), the wild-type and complemented strains both presented swimming behaviors on the semisolid swimming plates by 5.7 ± 0.1 cm and 5.1 ± 0.1 cm, respectively, compared to 3.7 ± 0.1 cm in the *gidA* inactivation mutant indicating a decreased in the swimming ability of the *gidA* mutant strain. In addition, the swarming plates revealed that the loss of *gidA* reduced the swarming ability of *P. aeruginosa* PA14 in covering the semisolid plate (Figure 3B). Furthermore, the lack of *gidA* was also found to affect biofilm production in *P. aeruginosa* PA14. Biofilm formation in the *gidA* mutant significantly decreased in relation to biofilm formation in the wild-type and complemented strains as determined by the dye staining method (Figure 3C). Biofilm formation was also observed using scanning electron microscope. The results demonstrated that *gidA* mutant was unable to form the confined biofilm compared to the wild-type and complemented strains (Figure 3D). In addition, alginate concentration was measured as it is an important component of biofilm (Gardner et al., 1987; Tatnell et al., 1994; Keiski et al., 2010). Consistent with the biofilm formation data, the alginate production was lower in the *gidA* mutant by 21.2 ± 1.8 folds. Taken together, these observations indicate that *gidA* contributes to various virulence genes in *P. aeruginosa* PA14, alter the flagellum-mediated motility activities (swimming and swarming) and biofilm formation.

### 3.4. The expression of some motility and biofilm formation genes gets altered in the *gidA* mutant

To further investigate the changes in the protein profile of the *gidA* mutant in comparison to the wild-type strain, proteomic analysis using liquid chromatography–tandem mass spectrometry was performed. A total of 1,106 protein identities were detected, 377 of which were differentially produced between the *gidA* mutant and wild-type strains (Supplementary Figure S2). Among these differentially produced proteins, 267 were upregulated and 110 were downregulated in the *gidA* mutant relative to the wild type. The abundance levels of several proteins involved in bacterial pathogenesis were altered (Table 3). A lower abundance of some components of the flagellar system, including FliD, FliS, and FliA, was seen in the *gidA* mutant than in the wild-type strain (Table 3). In addition, the levels of some proteins in the alginic acid biological process, which plays a role in biofilm formation, were found to decrease in the *gidA* mutant when compared with the levels in the wild-type strain; these included GDP-mannose dehydrogenase (AlgD) and multiple tetratricopeptide-like (TPR-like) repeats lipoprotein (AlgK). The level of MucA, an anti-sigma factor involved in the regulation of alginic acid biosynthesis, was higher in the *gidA* mutant than in the wild type. These data support the defects in mobility and biofilm formation observed in the *gidA* mutant.

**Table 3:** List of differentially produced proteins in *gidA* mutant compared to *Pseudomonas aeruginosa* wild type discussed in this report.

**Locus tag**	**Protein name**	**Accession number**	**Peptide sequence**^**1**^	**ID Score**^**2**^	**Log2 fold change**^**3**^
**Oxidative stress protection**					
PA14_61040	KatB/Catalase	gi|2,493,546	NLDPK	4.69	−0.72032
PA14_09150	KatA/Catalase	gi|6,647,442	GFSMR	7.84	−0.62002
**Flagella synthesis**					
PA14_45630	FliA or RpoF/Flagellar biosynthesis sigma factor	gi|120,310	GASFETYAGIRIR	2.68	−1.5171
PA14_50270	FliD/Flagellar capping protein	gi|13,124,216	MVNLEGAAK	11.21	−2.36944
PA14_50250	FliS/Flagellar protein	gi|14,548,039	GAMER	4.25	−2.8917
**Biofilm formation**					
PA14_18580	AlgD/GDP-mannose 6-dehydrogenase	gi|12,230,987	GYELR	1.62	−4.2173
PA14_18520	AlgK/Alginate biosynthetic protein	gi|33,300,924	AGRVPGER	5.65	−3.7617
PA14_54420	MucA/Anti-sigma factor	gi|585,528	WHEQR	2.59	2.51434

1 Peptide sequence; sequence of peptide that matches to the database with the highest ID score.

2 ID score; the highest score of the peptide sequence using Mascot software.

3 Analysis of variance (ANOVA) was used to assess significance (*p* ≤ 0.05).

**Figure 4:**
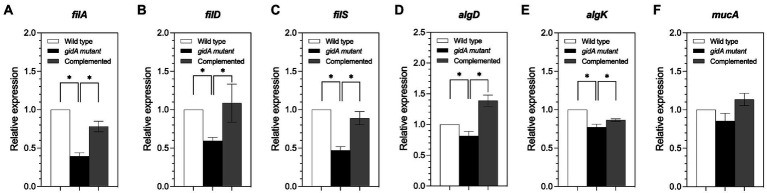
The *gidA* mutant strain shows defects in some motility and biofilm formation gene expression at the mRNA level. **(A)** Relative mRNA expression levels of *fliA*, **(B)** *fliD*, **(C)** *fliS*, **(D)** *algD*, **(E)** *algK*, and **(F)** *mucA* in the wild-type, *gidA* mutant, and complemented strains. The mRNA expressions of *fliA*, *fliD*, *fliS* (involved in motility), *algD*, and *algK* (involved in biofilm formation) genes were reduced in *gidA* mutant strains. The data represent the mean ± SD values of three biological replicates. The statistical analysis was performed using Graphpad Prism (GraphPad Software). Asterisks denote a significant difference in the one-way ANOVA (* *p* ≤ 0.05).

The mRNA levels of *fliA*, *fliD*, and *fliS*, which are involved in flagella formation, were investigated using semiquantitative RT-PCR analysis (Figure 4). The results revealed reduced mRNA levels in the *gidA* mutant strain compared to the wild-type and complemented strains (Figures 4A–C). With respect to genes involved in biofilm formation, the mRNA levels of the *algD* and *algK* genes were slightly lower in the *gidA* mutant strain than in the wild-type strain, while the mRNA level of the *mucA* gene remained unchanged in the *gidA* mutant but not in the wild-type strain (Figures 4D–F). The mRNA levels of the *algD* and *algK* genes changed in the same direction as the corresponding protein levels, indicating that such changes might not involve regulation at the translation level of those genes. The unchanged *mucA* mRNA level and upregulated protein level indicate the effect of *gidA* on *mucA* expression at the posttranscriptional level.

### 3.5. The *gidA* inactivation mutant attenuates virulence

**Figure 5:**
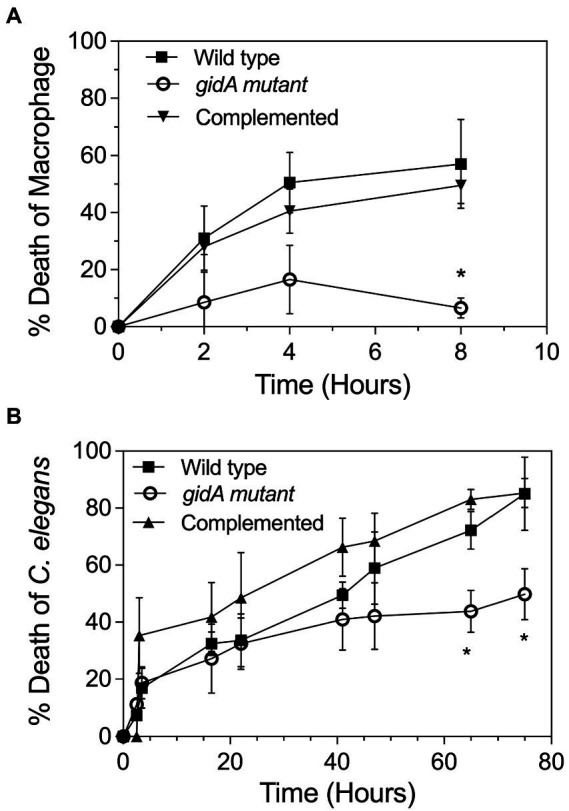
*gidA* is needed for the full virulence of *P. aeruginosa.* **(A)** Cell viability of the RAW264.7 macrophage cell line after treatment with the wild-type, *gidA* mutant, and complemented cells for 24 h. Cell viability at each timepoint was examined and compared with that of the untreated cells. **(B)** L4 stage larvae of *C. elegans* were added to the lawn of the wild-type, *gidA* mutant, and complemented cells. Live and dead worms were counted at time intervals for 3 days. The wild type and *gidA* mutant carried the pBBR1MCS-4 plasmid as a vector control. The data represent the mean ± SD values of three biological replicates. The statistical analysis was performed using Graphpad Prism (GraphPad Software). Asterisks denote a significant difference in the one-way ANOVA (* *p* ≤ 0.05).

In this study, the inactivation of *gidA* affected the oxidative stress response, motility, and biofilm formation capability of *P. aeruginosa* PA14 (Figures 2, 3), suggesting that the loss of *gidA* attenuates its virulence. Therefore, *in vitro* phagocytosis assays were performed using a mouse macrophage cell line (RAW264.7). The cells were infected with the wild-type, *gidA* mutant, and complemented strains of *P. aeruginosa* PA14 at a ratio of 10 bacterial cells per macrophage (MOI 1:10). Macrophages infected with the *gidA* inactivation mutant showed a significantly high survival rate compared to those infected with either the wild-type or complemented strains (Figure 5A). Further, the ability of the wild type, *gidA* inactivation mutant, and complemented strain to kill *Caenorhabditis elegans* was evaluated *in vivo* using a slow-killing assay. The results mirrored the macrophage assays. *P. aeruginosa* was clearly attenuated when *gidA* was disrupted. After 72 h of infection, the *C. elegans* fed with the *gidA* mutant had a significantly lower % of death (* *p* ≤ 0.05) than those fed with either the wild type or complemented strain (Figure 5B). Overall, these results confirmed that the inactivation of *gidA* attenuates the virulence of *P. aeruginosa* PA14.

## 4. Discussion

This study provided *in vivo* evidence that *P. aeruginosa gidA* functions as a tRNA-modifying enzyme involved in the biosynthetic pathway of mnm^5^(s^2^)U tRNA modification. Furthermore, the roles of *gidA* in the *P. aeruginosa* cellular response to oxidative stress, motility, biofilm production, and virulence were determined. The biosynthesis of 5-carboxymethylaminomethyl uridine (cmnm^5^U) and 5-methylaminomethyl uridine (mnm^5^U) tRNA modifications in *P. aeruginosa* were found to depend on both *gidA* and *trmE* (Figure 1B; Table 2; Supplementary Figure S1). The accumulation of s^2^U modification was detected in the total tRNA isolated from either the *gidA* and *trmE* mutant strains (Figure 1; Supplementary Figure S1), suggesting that the loss of either GidA or TrmE activity impairs the conversion of s^2^U to cmnm^5^(s^2^)U and/or to nm^5^(s^2^)U. According to the model for the biosynthetic pathway of mnm^5^(s^2^)U in *E. coli* (Moukadiri et al., 2014), the formation of cmnm^5^U in *P. aeruginosa* may be catalyzed by a GidA/TrmE complex *via* the glycine pathway (Figure 1A). The presence of nm^5^(s^2^)U in *P. aeruginosa* remains undetermined due to the unavailability of the standard. However, it is highly likely that the tRNAs of *P. aeruginosa* contain nm^5^(s^2^)U modifications. This is supported by the presence of the final product, mnm^5^U, in the wild-type tRNAs (Figure 1B) and the presence of *PA14_19400*, a homolog of *E. coli* MnmC in the genome of *P. aeruginosa.* MnmC is an enzyme that catalyzes the conversion of cmnm^5^(s^2^)U to nm^5^(s^2^)U.

The study results indicate that *gidA* contributes to H_2_O_2_ resistance in *P. aeruginosa*. In our previous studies, three tRNA modifying genes-*trmB*, *ttcA*, and *trmJ*-were shown to be associated with the H_2_O_2_ response of *P. aeruginosa* (Jaroensuk et al., 2016; Romsang et al., 2018; Thongdee et al., 2019). Among these three modifications, the molecular mechanism of the *trmB*-mediated H_2_O_2_ response and H_2_O_2_-dependent transcription regulation of *ttcA* have been described. The *trmB* gene, which encodes the tRNA m^7^G46 methyltransferase, modulates the H_2_O_2_ response by controlling the translation of *katA* and *katB* mRNAs *via* a bias codon usage mechanism (Thongdee et al., 2019). The *ttcA* gene, which encodes a tRNA thiolating enzyme, is directly regulated by OxyR, a transcription regulator that plays a global role in the oxidative stress response (Romsang et al., 2018). The observations in the present study suggest that the posttranscriptional regulation of *katA* and *katB* by tRNA modification is more complicated than we thought as *gidA* was also found to have a posttranscriptional impact on *katA* and *katB* expression (Figure 2; Table 3).

Furthermore, *gidA* is also involved in the posttranscriptional regulation of *phlA*, *phlD*, *rsmA*, and *rsmE* in *P. fluorescens* and *rhlR* in *P. aeruginosa* (Gupta et al., 2009; Zhang et al., 2014). The precise mechanism by which *gidA* selectively affects the translation of certain transcripts is unknown. Considering that the modification of wobble uridine by the GidA/TrmE complex ensures the discrimination of pyrimidine-ending codons from purine-ending codons and prevents translational frame shifting (Brégeon et al., 2001; Urbonavičius et al., 2001; El Yacoubi et al., 2012), the translation reduction in the *gidA* mutant strain may be a result of misreading problems.

In the present study, the *gidA* mutant showed significantly attenuated virulence in both the *C. elegans* model and the macrophage model (Figure 5). The disruption of *gidA* was found to reduce swimming, swarming, and biofilm formation, all of which contribute to bacterial virulence (Figures 3). These results are consistent with findings from previous studies that *gidA* deletion leads to decreased motility and biofilm formation in *Lysobacter capsica*, and virulence factor in *S. suis* and *Salmonella* (Rehl et al., 2013; Gao et al., 2016; Zhao et al., 2022). The proteomic study of *P. aeruginosa* also revealed that many proteins involved in motility and biofilm formation were downregulated in the *gidA* mutant compared to the wild type (Table 3). Bacterial flagella are essential for pathogenesis in *P. aeruginosa*, as they mediate swimming and swarming motility, bacterial adhesion, host invasion, and biofilm formation (Arora et al., 1998; Kirov, 2003). FliD, FliS and FliA, which are involved in flagellar biosynthesis, were found at lower levels in the *gidA* mutant (Table 3; Figure 4; Das et al., 2018; Bouteiller et al., 2021). This finding aligns with a previous report that a *fliS* mutant displays weak motility in *Yersinia pseudotuberculosis* (Xu et al., 2014). In addition, FliA, a positive regulator of cell motility, was slightly reduced in the *gidA* mutant. The above results imply that the depletion of *fliD*, *fliS*, and *fliA* genes at both mRNA and protein levels is involved in the reduced swimming and swarming motility levels in the *gidA* mutant of *P. aeruginosa*.

In the *gidA* mutant, the level of alginate was lower compared to wild type. The levels of AlgD, AlgK and MucA proteins were also altered (Table 3). These proteins are involved in the production of alginate, which is an important component of biofilm formation and is required for initial bacterial adhesion to a solid surface in *P. aeruginosa* (Gardner et al., 1987; Tatnell et al., 1994; Keiski et al., 2010). The levels of both AlgD and AlgK levels were downregulated in the *gidA* mutant. In contrast, MucA, an anti-AlgU sigma factor, was upregulated in the *gidA* mutant. Generally, MucA binds to AlgU, which is required for the transcription of the *algD* promoter. Thus, an increased MucA level might lead to reduced alginate biosynthesis due to AlgU down-regulation (Deretic et al., 1994). Interestingly, the mRNA level of *mucA* was unchanged in the *gidA* mutant but not in the wild-type strain indicating that *mucA* is subjected to post-transcription regulation by GidA.

In conclusion, the present study demonstrated that GidA is a tRNA-modifying enzyme of the mnm^5^U biosynthetic pathway. We revealed a novel role for *gidA* in the H_2_O_2_ stress response by demonstrating that *gidA* is required for full resistance against H_2_O_2_ and that it affects *katA*, *katB* and *mucA* expression at the posttranscriptional level. The results suggest that the posttranscriptional regulation of *katA* and *katB* by tRNA modification is a complex process involving multiple tRNA-modifying genes. In addition, *gidA* was shown to be involved in virulence of *P. aeruginosa*.

## Data availability statement

The data presented in the study are deposited in the ProteomeXchange repository (http://www.proteomexchange.org/, accession numbers JPST001884 and PXD037219.

## Author contributions

TS and NT contributed to the conception and design of the experiments, performed parts of the experiments, analyzed the results, and prepared the manuscript. JC performed the proteome data analysis and prepared the manuscript. SA performed the virulence assay. JJ, NP, and SR performed the mass spectrometry experiment. KP performed real-time PCR. SM was involved in the manuscript preparation. SA and KP performed SEM and alginate measurement. MF contributed to the conception and design of the experiments, analyzed the results, and prepared the manuscript. All authors contributed to the article and approved the submitted version.

## Funding

This work was supported by Chulabhorn Graduate Institute [ABS-01 to MF], by Thailand Science Research and Innovation (TSRI), Chulabhorn Research Institute (Grant no. 313/2231), and in part by the grant from Center of Excellence on Environmental Health and Toxicology (EHT), OPS, Ministry of Higher Education, Science, Research and Innovation. JC received Chulabhorn Graduate Scholarship and the Royal Golden Jubilee (RGJ) Ph.D. scholarship (Grant no. PHD/0196/2561) through the National Research Council of Thailand (NRCT), and Thailand Research Fund (TRF).

## Conflict of interest

The authors declare that the research was conducted in the absence of any commercial or financial relationships that could be construed as a potential conflict of interest.

## Publisher’s note

All claims expressed in this article are solely those of the authors and do not necessarily represent those of their affiliated organizations, or those of the publisher, the editors and the reviewers. Any product that may be evaluated in this article, or claim that may be made by its manufacturer, is not guaranteed or endorsed by the publisher.

## Footnotes

^1^https://repository.jpostdb.org/preview/12860057566342c8aa0471a
