# Supplementary material for: Pseudomonas aeruginosa GidA modulates the expression of catalases at the posttranscriptional level and plays a role in virulence
Source: Front Microbiol. 2023 Jan 16;13:1079710. doi: 10.3389/fmicb.2022.1079710 (PMC9884967; doi:10.3389/fmicb.2022.1079710)
Supplement: Supplementary file 1 [file Data_Sheet_1.PDF]

Supplementary Material

Pseudomonas aeruginosa **GidA modulates the expression of catalases at the posttranscriptional level and plays a role in virulence**

Thanyaporn Srimahaeak[1,2‡], Narumon Thongdee1[†‡], Jurairat Chittrakanwong[1], Sopapan Atichartpongkul[3], Juthamas Jaroensuk[1†], Kamonwan Phatinuwat[1], Narumon Phaonakrop[4], Janthima Jaresitthikunchai[4], Sittiruk Roytrakul[4], Skorn Mongkolsuk[1,3,5] and Mayuree Fuangthong[1,3,5 *]

[1]Program in Applied Biological Sciences, Chulabhorn Graduate Institute, Bangkok, Thailand, [2]Department of Biotechnology, Faculty of Engineering and Industrial Technology, Silpakorn University, Sanamchandra Palace Campus, Nakhon Pathom, Thailand, [3]Laboratory of Biotechnology, Chulabhorn Research Institute, Bangkok, Thailand, [4]Functional Ingredients and Food Innovation Research Group, National Center for Genetic Engineering and Biotechnology, National Science and Technology Development Agency, Pathum Thani, Thailand, [5]Center of Excellence on Environmental Health and Toxicology (EHT), OPS, MHESI, Bangkok, Thailand

‡These authors have contributed equally to this work.

* CORRESPONSENCE:

Mayuree Fuangthong

mayuree@cri.or.th

† PRESENT ADDRESSES:

Narumon Thongdee, Division of Molecular and Cellular Biology, Eunice Kennedy Shriver National Institute of Child Health and Human Development, National Institutes of Health, Bethesda, MD, United States

Juthamas Jaroensuk, School of Biomolecular Science and Engineering, Vidyasirimedhi Institute of Science and Technology (VISTEC), Rayong, Thailand

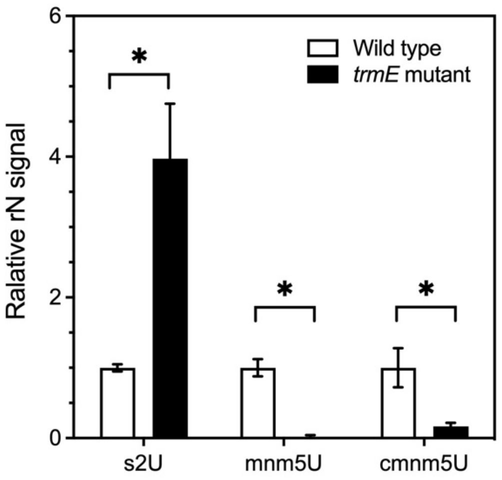

Supplementary Figure S1. The amount of tRNA modifications in mnm^5(s^2)U biosynthetic pathway is altered in the *trmE* mutant. Changes in the levels of s^{2}U, cmnm^{5}U, and mnm^{5}U in total tRNA isolated from the wild-type and *trmE* mutant strains. The wild type and *trmE* mutant carried the pBBR1MCS-4 plasmid as a vector control. The level of each modified ribonucleoside was quantified based on the MRM signal intensity and normalized by dividing the quantified amounts by the summed signals of adenosine, guanosine, cytidine, and uridine in the sample. The data represent the mean ± SD values of three biological replicates. The statistical analysis was performed using Graphpad Prism (GraphPad Software). Asterisks denote a significant difference in the one-way ANOVA (* $p \leq 0.05$).

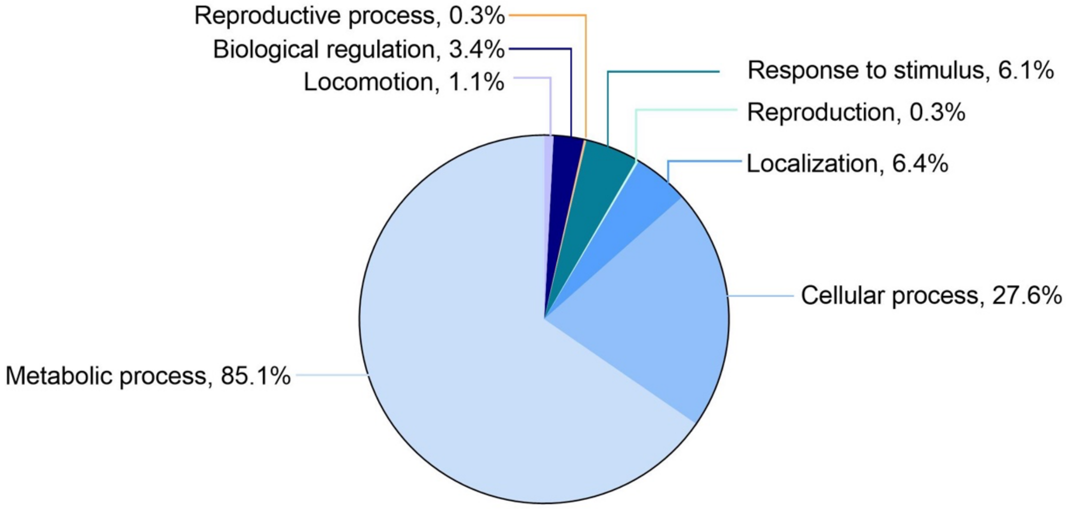

Supplementary Figure S2. Categories of differentially produced proteins in the wild type and *gidA* mutant according to the Gene Ontology Annotation Database. The 377 upregulated and downregulated proteins identified were classified based on their biological processes. The functions of the identified proteins could be classified as cellular process (104, 27.6%), metabolic process (321, 85.1%), response to stimulus (23, 6.1%), biological regulation (13, 3.4%), localization (24, 6.4%), locomotion (4, 1.1%), reproduction (1, 0.3%), or reproductive process (1, 0.3%).
